# D‐tryptophan, an eco‐friendly natural, safe, and healthy compound with antimicrobial activity against food‐borne pathogens: A systematic review

**DOI:** 10.1002/fsn3.3987

**Published:** 2024-02-01

**Authors:** Minoo Moghimani, Seyyed Mohammad Ali Noori, Asma Afshari, Mohammad Hashemi

**Affiliations:** ^1^ Medical Toxicology Research Center Mashhad University of Medical Sciences Mashhad Iran; ^2^ Department of Nutrition, Faculty of Medicine Mashhad University of Medical Sciences Mashhad Iran; ^3^ Toxicology Research Center Medical Basic Sciences Research Institute, Ahvaz Jundishapur University of Medical Sciences Ahvaz Iran; ^4^ Department of Nutrition, School of Allied Medical Sciences Ahvaz Jundishapur University of Medical Sciences Ahvaz Iran

**Keywords:** antibiofilm activity, antimicrobial activity, dairy products, D‐tryptophan, food‐borne pathogens, rice pudding, seafood

## Abstract

Recently, the use of D‐amino acids as food preservatives has attracted considerable attention because these natural compounds do not have adverse effects on human health. In addition, D‐amino acids such as D‐tryptophan can reduce the harmful effects of other treatments. For instance, the use of D‐tryptophan in food reduces the requirement for high temperatures and their damaging effects on nutrients such as proteins and vitamins. The purpose of this systematic review was to investigate the antimicrobial effect of D‐tryptophan on food‐borne pathogens in vitro and in food models. To identify related studies, scientific digital databases such as PubMed, Science Direct, and Google Scholar were searched from January 2000 to February 2023. The results of the studies showed that when D‐tryptophan was used with other stresses such as using different salt concentrations, refrigeration, or high temperatures, it showed significant antimicrobial effects on Gram‐positive and Gram‐negative food‐borne pathogens, and antibiofilm impacts were also observed with D‐tryptophan. Since studies have shown that the antimicrobial activity of D‐tryptophan depends on several factors, including the pathogen strain, the type of stress, and the concentration of D‐tryptophan, and every article has focused on one of these factors, there is a need for a systematic review that summarizes and concludes the effect of all these factors on the antimicrobial activity of D‐tryptophan against food‐borne pathogens.

## Graphical abstract

In general, it can be said that D‐tryptophan has an antimicrobial effect and can be used as a natural preservative in food. The genus and species of bacteria, osmotic stress, temperature stress and D‐tryptophan concentration are among the factors that affect the antimicrobial activity of D‐tryptophan. D‐tryptophan also has antibiofilm activity in the laboratory environment.

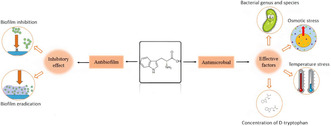

## 1 INTRODUCTION

Maintaining food safety, especially for foods that are consumed raw or not sufficiently processed (e.g. ready‐to‐eat foods), is considered a serious public health concern. Despite the great advances in food technology, hospitalizations due to food‐borne pathogens are reported in approximately 300,000 people, which leads to 5000 deaths annually. In addition, its economic loss is estimated at 7 billion dollars; therefore, food safety departments, food experts, and regulatory organizations must pay special attention to this issue (Brandão et al., 2023; Elafify, Darwish, et al., 2022; Elafify, Sadoma, et al., 2022; Liu et al., 2019).

On the other hand, synthetic antimicrobial compounds are not only harmful to human health but may also cause off‐flavors in foods. Moreover, the excessive use of these compounds has caused bacterial resistance (Afshari et al., 2022; Batiha et al., 2021; Chen et al., 2022). Therefore, the demand for healthy, natural, and high‐quality processed foods is growing rapidly. One solution to reduce the use of chemical preservatives is to replace them with bio‐preservatives (Elafify, Sadoma, et al., 2022; Mith et al., 2014; Moghimani et al., 2023; Mohan et al., 2021).

Bio‐preservatives are produced naturally by plants, animals, and bacteria (Batiha et al., 2021; Liang et al., 2019; Moghimani et al., 2023; Salimnejhad et al., 2023). In this regard, different studies were conducted to find natural and safe antimicrobial compounds to be used in the food industry (Elafify et al., 2019; Erfani et al., 2023; Touranlou et al., 2023). D‐amino acids are a group of these antimicrobial compounds (Martínez‐Rodríguez et al., 2010).

D‐amino acids are enantiomers of L‐amino acids, which were considered non‐functional for a long time. However, further studies discovered that some D‐amino acids, which are in mammalian tissues, contribute to critical physiological activities (Seki et al., 2022).

D‐tryptophan is one of these D‐amino acids, and studies on it have approved its antimicrobial properties. For instance, Koseki et al. (2015) reported that D‐arginine and D‐proline had a mild antimicrobial effect at 40 mM and ≥3% NaCl, whereas D‐tryptophan had a more significant antimicrobial effect under similar conditions (more than a 12‐h delay in the growth of various pathogens) (Comai et al., 2020; Koseki et al., 2015).

Another characteristic of D‐tryptophan is its antibiofilm activity. Biofilms are communities formed by one or more bacterial species in an extracellular matrix (Li et al., 2015; Rezaei et al., 2023). The formation of biofilms causes several problems. For instance, biofilms participate in the pathogenesis and transmission of food‐borne pathogens. Moreover, in the food industry, biofilms cause microbiologically influenced corrosion (MIC), equipment failure, economic losses, and environmental damage (Elgamoudi et al., 2020; Jia, Li, et al., 2017; Jia, Yang, et al., 2017; Wu et al., 2015). Studies have shown that D‐tryptophan alone or in combination with a mixture of other D‐amino acids can have antibiofilm properties (Brandenburg et al., 2013; Elgamoudi et al., 2020; Ghosh et al., 2019; Jayalekshmi et al., 2016; Jia, Li, et al., 2017; Jia, Yang, et al., 2017; Kolodkin‐Gal et al., 2010; Leiman et al., 2013; Li et al., 2015; Zilm et al., 2017).

The mechanism of antimicrobial and anti‐biofilm activity of D‐tryptophan has not yet been fully understood, but it seems that these functional properties are due to the prevention of initial cell adhesion caused by changes in the extracellular matrix (Elafify et al., 2019; Elafify, Sadoma, et al., 2022).

Since studies have shown that the antimicrobial activity of D‐tryptophan depends on several factors, including the strain of the pathogen, the type of stress, and the concentration of D‐tryptophan, and every article has focused on one of these factors, there is a need for a systematic review that summarizes and concludes the effect of all these factors on the antimicrobial activity of D‐tryptophan against food‐borne pathogens.

## 2 METHOD

**FIGURE 1:**
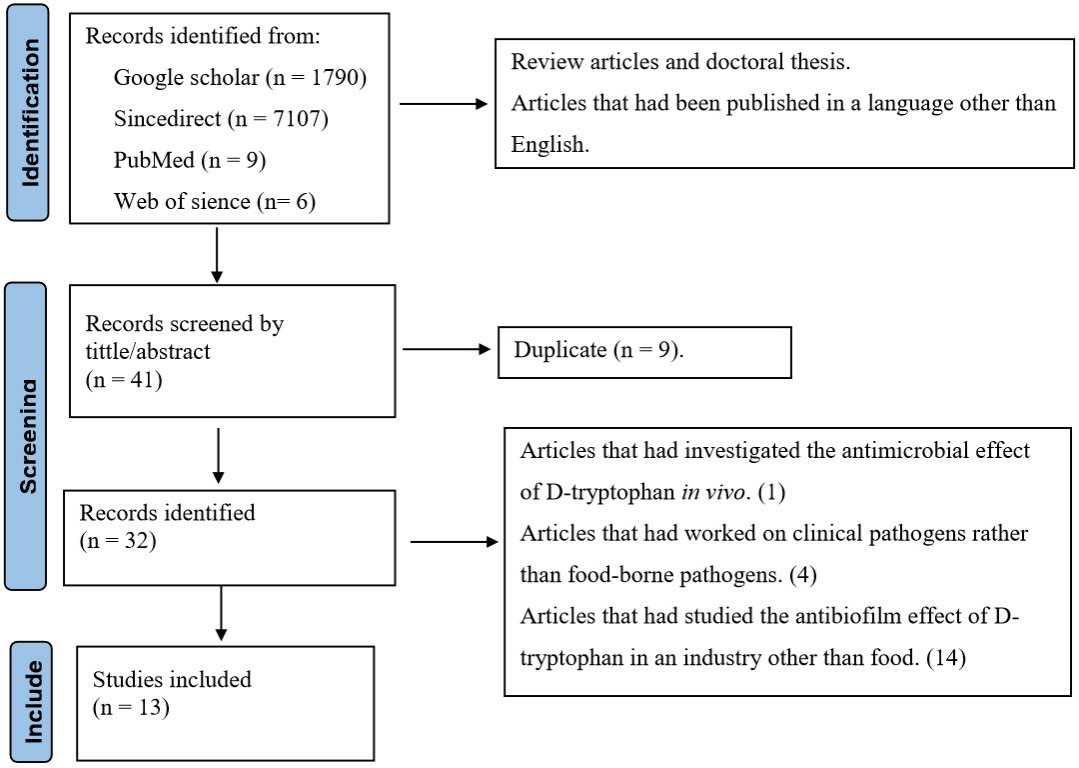
Flow diagram of the study selection process.

This article is a systematic review based on PRISMA (Preferred Reporting Items for Systematic Reviews and Meta‐analysis) (Figure 1) to explore the effect of the antimicrobial activity of D‐tryptophan on food‐borne pathogens in vitro and in food models.

### 2.1 Search strategy

To identify relevant studies, scientific digital databases such as PubMed, Science Direct, Web of Science, and Google Scholar were searched from January 2000 to February 2023. A total of 8912 articles were collected from all databases, 13 of which met the inclusion criteria (Figure 1).

The query was: ((D‐tryptophan) AND (antimicrobial) AND (food)).

### 2.2 Data eligibility, article selection, and result synthesis

The articles were screened by MM (Minoo Moghimani) based on their titles and abstracts and were then re‐examined by SN (Seyyed Mohammad Ali Noori), AA (Asma Afshari), and MH (Mohammad Hashemi). Articles that did not meet the inclusion criteria were removed. Finally, the full text of the related articles was assessed.

### 2.3 Inclusion and exclusion criteria

The included articles should investigate the antimicrobial and antibiofilm effects of D‐tryptophan on food‐borne pathogens, either in vitro or in food models, and should have been published in English. The exclusion criteria were review articles, doctoral thesis, articles that had been published in a language other than English, articles that had investigated the antimicrobial effect of D‐tryptophan in vivo, articles that had worked on clinical pathogens rather than food‐borne pathogens, and articles that had studied the antibiofilm effect of D‐tryptophan in an industry other than food.

### 2.4 Risk of bias assessment

Different sections of the included articles were qualitatively reviewed by the authors to assess the risk of bias. The results of this evaluation are shown in different colors, such as green (good), yellow (moderate), and red (poor), in Table 1.

**TABLE 1:** Table of risk of bias assessment.

Study ID (reference)	Risk of biasness parameters					
	Research rationale	Description of methods	Characterization and testing	Description of results	discussion	Overall conclusion
Elafify (2022)						
Chen (2018)						
Chen (2020)						
Chen (2022)						
Elafify (2020)						
Elafify (2019)						
Elafify, Darwish, et al. (2022)						
Elafify, Sadoma, et al. (2022)						
Elafify (2023)						
Kan (2018)						
Koseki (2015)						
Elgamoudi (2020)						
Li (2015)						

*Note*: The results of risk of bias assessment are shown in different colors, such as green (good), yellow (moderate), and red (poor).

## 3 RESULTS AND DISCUSSION

Studies have shown that D‐tryptophan is an antimicrobial compound that is not solely effective against all pathogens and must be used with other stresses (Chen et al., 2018, 2020, 2022; Elafify et al., 2019, 2020, 2023; Elafify, Darwish, et al., 2022; Elafify, Sadoma, et al., 2022; Elgamoudi et al., 2020; Koseki et al., 2015; Li et al., 2015; Seki et al., 2022).

Although the mechanism of antimicrobial activity of D‐tryptophan is still not well known, it appears that D‐tryptophan reduces the initial adhesion of pathogenic cells and changes the characteristics of the extracellular environment, which have a significant effect on the weakening of pathogenic cells. D‐tryptophan also prevents the formation of biofilms by using the same mechanism and acts as an autoinhibitory compound, which prevents the germination of spores and the growth of pathogens (Elafify et al., 2019, 2023; Elafify, Sadoma, et al., 2022).

**FIGURE 2:**
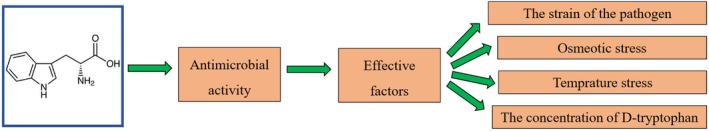
Effective factors on the antimicrobial activity of D‐tryptophan.

According to studies, several factors affect the antimicrobial activity of D‐tryptophan, as shown in Figure 2.

### 3.1 The strain of the pathogen

The results of the studies revealed that the antimicrobial effect of D‐tryptophan is strain‐dependent. For instance, Chen et al. (2022) reported that although D‐tryptophan had an antimicrobial effect on different strains of *Listeria monocytogenes*, the survival rates of the strains were different (Chen et al., 2022). Similar results were reported by Chen et al. (2018), Elafify, Darwish, et al. (2022), Koseki et al. (2015).

### 3.2 Osmotic stress

The results regarding the antimicrobial effect of D‐tryptophan, along with osmotic stress on different food‐borne pathogens, are summarized in Table 2. These results showed that co‐administration of D‐tryptophan and osmotic stress inhibited the growth of food‐borne pathogens more than the control (without D‐tryptophan) in all studies (Chen et al., 2018, 2022; Kan et al., 2018; Koseki et al., 2015; Radoshevich & Cossart, 2018).

**TABLE 2:** Studies conducted on the antimicrobial effect of D‐tryptophan along with osmotic stress on food‐borne pathogens.

Author	Year	Concentration of D‐Trp	Medium	Pathogen	Temperature	Method	Concentration of component	Component	Effect	Reduction	Time	Ref
Chen	2022	15 mM	LB broth	*P. fluorescens S. baltica*	28°C	Optical density at 595 nm	5%	NaCl	Yes	–	5 days	Chen et al. (2022)
Chen	2018	40 mM	TSB	*V. parahemolyticus/ V. vulnificus*	25°C	Optical density at 595 nm	>4.0%	NaCl	Yes	>4.0 log CFU/mL	3 days	Chen et al. (2018)
Kan	2018	40 mM	PYG Broth	*E. coli*	25°C	Optical density at 595 nm	>2.5%	NaCl	Yes	–	5 days	Kan et al. (2018)
Kan	2018	35 mM	PYG Broth	*E. coli*	25°C	Optical density at 595 nm	3%	NaCl	Yes	–	5 days	Kan et al. (2018)
Kan	2018	40 mM	PYG Broth	*E. coli*	25°C	Optical density at 595 nm	58.60%	Sucrose	No	–	5 days	Kan et al. (2018)
Kan	2018	40 mM	PYG Broth	*E. coli*	25°C	Optical density at 595 nm	6.30%	KCl	Yes	–	5 days	Kan et al. (2018)
Koseki	2015	40 mM	PYG Broth	*E. coli 0 1 57:H7*	25°C	Optical density at 595 nm	>3%	NaCl	Yes	>12‐h delay of growth	40 h	Koseki et al. (2015)
Koseki	2015	40 mM	PYG Broth	*S. enterica*	25°C	Optical density at 595 nm	>3%	NaCl	Yes	>12‐h delay of growth	40 h	Koseki et al. (2015)

The hypothesis justifying the effect of osmotic stress on the antimicrobial activity of D‐tryptophan is that bacterial cells absorb a set of compatible solutes, such as glycine betaine, to manage osmotic stress. Therefore, the co‐administration of osmotic stress and D‐tryptophan causes more absorption of D‐tryptophan by the bacterial cell, which is an incompatible compound, and subsequently causes disturbances in cell metabolism (Koseki et al., 2015).

Kan et al. (2018) investigated the effect of ionic (sodium chloride and potassium chloride) and molecular (sugar) compounds on creating the osmotic pressure, which showed that the effective osmotic pressure with D‐tryptophan to reduce *E. coli* was 2.4 MPa. They also reported that sugar, in amounts of 58.6%, created an osmotic pressure of 2.4 MPa, but together with D‐tryptophan had no inhibitory effect, whereas potassium chloride and sodium chloride, with concentrations of 6.3% and 3% and osmotic pressure of 2.4 Mpa, had a weak inhibitory effect and a remarkable inhibitory effect with D‐tryptophan, respectively (Table 2). Therefore, it can be concluded that the use of ionic components, especially sodium chloride, is more suitable for creating effective osmotic pressure because its small amounts can create enough osmotic pressure with D‐tryptophan, which inhibits the growth of pathogens (Kan et al., 2018).

### 3.3 Temperature stress

The results regarding the antimicrobial effects of D‐tryptophan and temperature stress on different food‐borne pathogens have been summarized in Table 3. These results indicate that co‐administration of D‐tryptophan and low temperature (refrigeration) have a better antimicrobial effect compared to the control (without D‐tryptophan). Chen et al. (2022) showed that *Shivanella baltica* and *Pseudomonas fluorescens*, without D‐tryptophan, tolerated refrigeration, and the presence of D‐tryptophan could effectively reduce their growth (Chen et al., 2022).

**TABLE 3:** Studies conducted on the antimicrobial effect of D‐tryptophan along with temperature stress on food‐borne pathogens.

Author	Year	Concentration of D‐Trp	Medium	Pathogen	Temperature	Method	Effect	Reduction	Time	Ref
Elafify	2023	40 mM	PYG Broth	*E. coli O26:H11*	4°C	Survival bacterial count	Yes	1.5 log CFU/mL	4 weeks	Elafify et al. (2023)
Chen	2022	40 mM	LB broth	*S. baltica*	4°C	Survival bacterial count	Yes	0.3 log CFU/mL	5 days	Chen et al. (2022)
Chen	2022	40 mM	LB broth	*P. fluorescens*	4°C	Survival bacterial count	Yes	0.5 log CFU/mL	5 days	Chen et al. (2022)
Chen	2020	40 mM	PYG	*L. monocytogenes*	4°C	Optical density at 595 nm	Yes	Growth inhibition	400 h	Chen et al. (2020)
Chen	2020	40 mM	PYG	*L. monocytogenes*	37°C	Optical density at 595 nm	No	0	400 h	Chen et al. (2020)
Kan	2018	40 mM	TSA	*E. coli*	43°C	Survival bacterial count	Yes	4.5 log CFU/mL	5 days	Kan et al. (2018)
Kan	2018	40 mM	TSA	*E. coli*	46°C	Survival bacterial count	Yes	4.5 log CFU/mL	3 days	Kan et al. (2018)
Kan	2018	40 mM	TSA	*E. coli*	37°C	Survival bacterial count	Yes	4 log CFU/mL	5 days	Kan et al. (2018)
Kan	2018	40 mM	TSA	*E. coli*	25°C	Survival bacterial count	Yes	2.5 log CFU/mL	5 days	Kan et al. (2018)
Kan	2018	40 mM	TSA	*E. coli*	15°C	Survival bacterial count	Yes	2 log CFU/mL	5 days	Kan et al. (2018)

Kan et al. (2018) reported that D‐tryptophan does not require high temperatures to eliminate pathogens. In this study, the presence of D‐tryptophan at a temperature of 43–45°C reduced the number of *E. coli* below the detection limit (4.5 log cfu/mL reduction). In contrast, without D‐tryptophan, higher temperatures were required to reduce *E. coli* (Kan et al., 2018). Therefore, the use of D‐tryptophan, by reducing the temperature required to destroy pathogens, prevents the adverse effects of high temperatures on protein structure and the performance of essential amino acids and vital nutrients in foods (Abraha et al., 2018).

**FIGURE 3:**
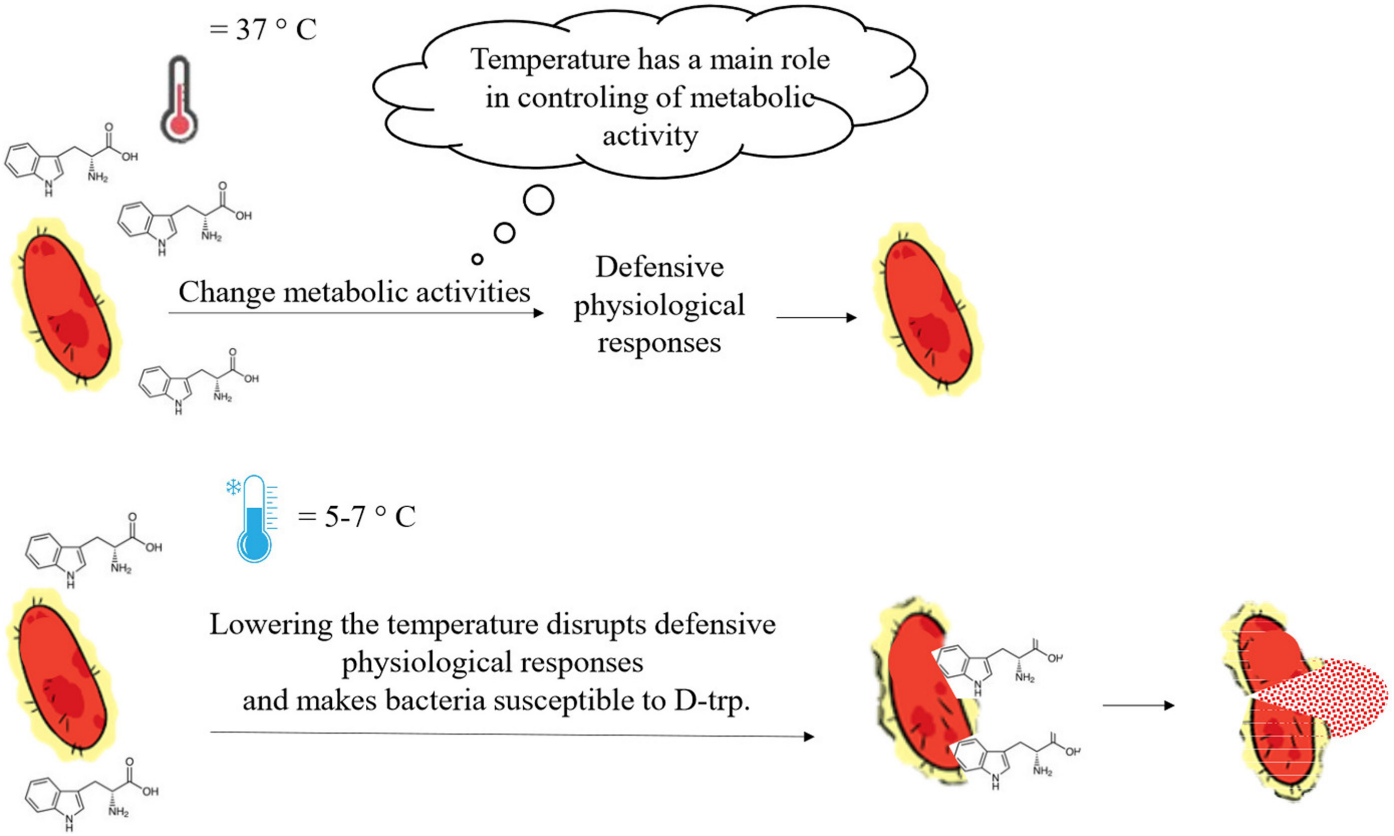
Mechanism of co‐administration of D‐tryptophan and temperature stress. In general, when the bacterial cell is exposed to D‐tryptophan, it creates physiological defensive responses through changing metabolic activities to protect itself, and temperature is one of these controlling factors; therefore, when the temperature changes, physiological defensive responses are disturbed, and the bacterial cell becomes vulnerable to D‐tryptophan.

The mechanism of the effect of administration of D‐tryptophan and temperature stress is such that when the bacterium is exposed to D‐tryptophan as an environmental stress, it produces a series of defensive physiological responses by changing its metabolic activity, and protects the bacterial cell against D‐tryptophan. One of the control factors of the change in metabolic activity is temperature, and changes in temperature lead to dysfunctions in the development of physiological defense responses, and finally, the bacterial cell becomes vulnerable to D‐tryptophan (Figure 3) (Kan et al., 2018).

### 3.4 Concentration of D‐tryptophan

The results of the studies suggested that the concentration of D‐tryptophan is another effective factor in the antimicrobial activity of D‐tryptophan. The results also showed that the ideal concentration is 40 mM, and concentrations below 40 mM do not have an acceptable inhibitory effect (Chen et al., 2020; Elafify, Sadoma, et al., 2022).

### 3.5 Use of D‐tryptophan in food

D‐tryptophan is a safe natural compound, and in vitro studies have shown that D‐tryptophan, along with other stresses including osmotic and temperature, has good antimicrobial activity, which makes it an appropriate natural food preservative (Chen et al., 2020).

Various studies have investigated the antimicrobial effect of D‐tryptophan on food‐borne pathogens in different food models. The results shown in Table 4 indicate the antimicrobial effect of D‐tryptophan on *L. Monocytogenes* were more significant in fresh heavy cream compared to milk, probably due to its lower water activity and higher fat content, which create conditions similar to osmotic stress (Elafify et al., 2020).

**TABLE 4:** Studies conducted on the use of D‐tryptophan as a natural preservative along with several stressful conditions in food.

Author	Year	Food	Concentration of D‐Trp	Pathogen	Temperature	Component	Concentration of component	Method	Effect	Reduction	Time	Ref
Elafify	2022	Soft cheese	40 mM	*E. coli O26:H11*	4°C	NaCl	1.50%	Survival bacterial count	Yes	1 log CFU/g	4 weeks	Elafify, Sadoma, et al. (2022)
Elafify	2022	Soft cheese	40 mM	*E. coli O26:H11*	4°C	NaCl	3%	Survival bacterial count	Yes	1.5 log CFU/g	2 weeks	Elafify, Sadoma, et al. (2022)
Elafify	2022	Soft cheese	40 mM	*E. coli O26:H11*	4°C	NaCl	3%	Survival bacterial count	Yes	2.5 log CFU/g	4 weeks	Elafify, Sadoma, et al. (2022)
Elafify	2022	Ice cream	40 mM	*E. coli O26:H11*	−20°C	NaCl	0	Survival bacterial count	Yes	1.7 log CFU/g	2 weeks	Elafify, Sadoma, et al. (2022)
Elafify	2022	Ice cream	40 mM	*E. coli O26:H11*	−20°C	NaCl	0	Survival bacterial count	Yes	2 log CFU/g	4 weeks	Elafify, Sadoma, et al. (2022)
Chen	2018	Shucked oysters immersed in peptone water	40 mM	*V. parahaemolyticus*, *V. vulnificus*	25°C	NaCl	3.5%, 4.0%, 4.5%, and 5.0%	Survival bacterial count	Yes	–	48 h	Chen et al. (2018)
Chen	2018	Shucked oysters immersed in artificial seawater	40 mM	*V. parahaemolyticus*, *V. vulnificus*	25°C	NaCl	3.5%, 4.0%, 4.5%, and 5.0%	Survival bacterial count	Yes	–	3 days	Chen et al. (2018)
Chen	2018	Live oysters with shells	40 mM	*V. vulnificus*	25°C	NaCl	0	Survival bacterial count	Yes	2.9 log CFU/g	48 h	Chen et al. (2018)
Chen	2018	Live oysters with shells	40 mM	*V. parahaemolyticus*	25°C	NaCl	0	Survival bacterial count	Yes	2.2 log CFU/g	48 h	Chen et al. (2018)
Chen	2018	Shucked oysters immersed in artificial seawater	40 mM	*V. parahaemolyticus*, *V. vulnificus*	4°C	NaCl	3.50%	Survival bacterial count	Yes	No change of initial value	3 days	Chen et al. (2018)
Chen	2018	Shucked oysters immersed in artificial seawater	40 mM	Total bacterial population	4°C	NaCl	5%	Survival bacterial count	Yes	No change of initial value	5 days	Chen et al. (2018)
Chen	2020	Pasteurized milk	40 mM	Different strains of *L. monocytogenes*	4°C	NaCl	0	Survival bacterial count	Yes	>3.5 log CFU/mL	1 month	Chen et al. (2020)
Chen	2020	Raw milk	40 mM	Total psychrotrophic bacteria	4°C	NaCl	0	Survival bacterial count	Yes	No change of initial value	3 days	Chen et al. (2020)
Chen	2022	Salmon fillets	40 mM	Total viable bacteria	4°C	NaCl	3.50%	Total viable bacteria count	Yes	No change of initial value	2 days	Chen et al. (2022)
Elafify	2020	Milk	40 mM	Different strains of *L. monocytogenes*	4°C	NaCl	0	Survival bacterial count	Yes	1.5 log CFU/mL	2 weeks	Elafify et al. (2020)
Elafify	2020	Milk	40 mM	Different strains of *L. monocytogenes*	4°C	NaCl	0	Survival bacterial count	Yes	–	4 weeks	Elafify et al. (2020)
Elafify	2020	Milk	40 mM	Different strains of *L. monocytogenes*	7°C	NaCl	0	Survival bacterial count	Yes	2 log CFU/mL	2 weeks	Elafify et al. (2020)
Elafify	2020	Milk	40 mM	Different strains of *L. monocytogenes*	7°C	NaCl	0	Survival bacterial count	Yes	–	4 weeks	Elafify et al. (2020)
Elafify	2020	Milk	40 mM	Different strains of *L. monocytogenes*	10°C	NaCl	0	Survival bacterial count	Yes	1 log CFU/mL	1 week	Elafify et al. (2020)
Elafify	2020	Milk	40 mM	Different strains of *L. monocytogenes*	10°C	NaCl	0	Survival bacterial count	Yes	1.4 log CFU/mL	2 weeks	Elafify et al. (2020)
Elafify	2020	Milk	40 mM	Different strains of *L. monocytogenes*	10°C	NaCl	0	Survival bacterial count	Yes	0.9 log CFU/mL	3 weeks	Elafify et al. (2020)
Elafify	2020	Milk	40 mM	Different strains of *L. monocytogenes*	10°C	NaCl	0	Survival bacterial count	Yes	0.8 log CFU/mL	4 weeks	Elafify et al. (2020)
Elafify	2020	Milk	40 mM	Different strains of *L. monocytogenes*	55°C	NaCl	0	Survival bacterial count	Yes	0.6 log CFU/mL	5 min	Elafify et al. (2020)
Elafify	2020	Milk	40 mM	Different strains of *L. monocytogenes*	55°C	NaCl	0	Survival bacterial count	Yes	0.6 log CFU/mL	10 min	Elafify et al. (2020)
Elafify	2020	Milk	40 mM	Different strains of *L. monocytogenes*	55°C	NaCl	0	Survival bacterial count	Yes	2 log CFU/mL	15 min	Elafify et al. (2020)
Elafify	2020	Milk	40 mM	Different strains of *L. monocytogenes*	55°C	NaCl	0	Survival bacterial count	Yes	1.2 log CFU/mL	20 min	Elafify et al. (2020)
Elafify	2020	Milk	40 mM	Different strains of *L. monocytogenes*	55°C	NaCl	0	Survival bacterial count	Yes	1.6 log CFU/mL	25 min	Elafify et al. (2020)
Elafify	2020	Milk	40 mM	Different strains of *L. monocytogenes*	55°C	NaCl	0	Survival bacterial count	Yes	1.7 log CFU/mL	30 min	Elafify et al. (2020)
Elafify	2020	Milk	40 mM	Different strains of *L. monocytogenes*	60°C	NaCl	0	Survival bacterial count	Yes	No L. monocytogenes was detected	25 min	Elafify et al. (2020)
Elafify	2020	Milk	40 mM	Different strains of *L. monocytogenes*	65°C	NaCl	0	Survival bacterial count	Yes	No L. monocytogenes was detected	20 min	Elafify et al. (2020)
Elafify	2020	Heavy fresh cream	40 mM	Different strains of *L. monocytogenes*	4°C, 7°C, 10°C	NaCl	0	Survival bacterial count	Yes	‐	4 weeks	Elafify et al. (2020)
Elafify	2019	UHT milk	40 mM	Different strains of *S. typhimurium*	50°C	NaCl	0	Survival bacterial count	Yes	1.85 log CFU/mL	10 min	Elafify et al. (2019)
Elafify	2019	UHT milk	40 mM	Different strains of *S. typhimurium*	55°C	NaCl	0	Survival bacterial count	Yes	1.97 log CFU/mL	10 min	Elafify et al. (2019)
Elafify	2019	UHT milk	40 mM	Different strains of *S. typhimurium*	60°C	NaCl	0	Survival bacterial count	Yes	2.3 log CFU/mL	10 min	Elafify et al. (2019)
Elafify	2022	Soft cheese	40 mM	*S. enteritidis*	4°C	NaCl	1.50%	Survival bacterial count	Yes	1 log CFU/mL	4 weeks	Elafify, Darwish, et al. (2022)
Elafify	2022	Soft cheese	40 mM	*S. enteritidis*	4°C	NaCl	3%	Survival bacterial count	Yes	2.1 log CFU/mL	4 weeks	Elafify, Darwish, et al. (2022)
Elafify	2023	Rice pudding	40 mM	Three different strains of *B. cereus*	4°C	Sugar	30%	Spore counts	Yes	1.2 log CFU/g	4 weeks	Elafify et al. (2023)
Elafify	2023	Rice pudding	40 mM	Three different strains of *B. cereus*	4°C	Sugar	50%	Spore counts	Yes	1.8 log CFU/g	4 weeks	Elafify et al. (2023)
Elafify	2023	Rice pudding	40 mM	Three different strains of *B. cereus*	10°C	Sugar	30%	Spore counts	Yes	1.1 log CFU/g	4 weeks	Elafify et al. (2023)
Elafify	2023	Rice pudding	40 mM	Three different strains of *B. cereus*	10°C	Sugar	50%	Spore counts	Yes	1.7 log CFU/g	4 weeks	Elafify et al. (2023)

Elafify et al. (2023) used D‐tryptophan for the first time as an anti‐spore additive in rice pudding, which could effectively prevent the growth of *Bacillus cereus* spores. The anti‐sporogenesis mechanism of D‐tryptophan is due to the autoinhibition phenomenon, which inhibits germination and prevents the growth of spores (Elafify et al., 2023).

Chen et al. (2018) investigated the antimicrobial effect of 40 mM D‐tryptophan on respiratory active cells of *S. baltica* and *P. fluorescens* in marine products at 1%–3% NaCl and 4°C. In this study, the number of cells decreased gradually, and the most significant reduction was observed at 3% NaCl concentration. The importance of respiratory active cells is due to their ability to aerobically spoil marine products because their number determines their spoilage potential. In fact, by preventing the growth of respiratory active cells, D‐tryptophan delays the spoilage process in marine products (with a high spoilage rate) (Chen et al., 2018).

Chen et al. (2022) also evaluated the effect of D‐tryptophan at 3.5% NaCl and 4°C on the amount of total volatile basic nitrogen (TVB‐N) in fish fillets. The initial amount of TVB‐N in fish fillets was 8.0 mg/100 g. It reached 30 mg N/100 g in the control (without D‐tryptophan) on the eighth day, which is considered rotten fish. However, with 40 mM D‐tryptophan, the amount of TVB‐N reached below 30 mg N/100 g on day 10 of the storage. The amount of TVB‐N is considered an indicator of spoilage in marine products, and its reduction by D‐tryptophan may be due to the impact of D‐tryptophan on reducing the population of spoilage microorganisms or their metabolic activity in the oxidative deamination of non‐protein nitrogen compounds (Chen et al., 2022).

Elafify et al. (2019) investigated the impact of 40 mM D‐tryptophan on the D‐value. In this study, the D‐value in the control (without D‐tryptophan) at 50, 55, and 60°C was 25.05, 16, and 10.57 min, respectively. With D‐tryptophan, the D‐value was 12.28, 11.27, and 8.32 min, respectively (Elafify et al., 2019). It was similar to the results obtained by Elafify et al. (2020). In this article, the D‐value in control (without D‐tryptophan) at 55, 60, and 65°C was 200, 97, and 86 min, respectively. While with 40 mM of D‐tryptophan, it was 136, 75, and 75 min. These studies showed that the presence of D‐tryptophan reduced the D‐value, which also reduced the negative effect of high temperatures on nutrients (Elafify et al., 2020).

### 3.6 Antibiofilm activity of D‐tryptophan

Some bacteria can adapt to harsh chemical and physical conditions by forming a matrix consisting of exopolysaccharides, proteins, and extracellular DNA called biofilm in the environment (Sarkar & Pires, 2015). The capacity of food‐borne pathogens to create biofilms is the main virulence factor in their pathogenesis and transmission. For instance, the formation of biofilm by *Campylobacter jejuni*, a food‐borne pathogen, facilitates its transmission through the food chain. Unfortunately, there are no solutions to prevent it (Elgamoudi et al., 2020; Li et al., 2015). In addition, biofilms can damage both industrial equipment and the environment and, in some cases, can cause irreparable damage (Jia, Yang, et al., 2017).

**FIGURE 4:**
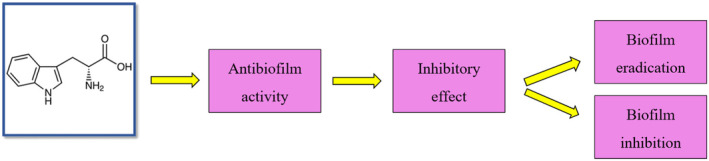
Mechanism of antibiofilm activity of D‐tryptophan.

In most cases, the only solution to solve this problem is to replace biofilm‐contaminated equipment with new ones, which can be expensive and life‐threatening. Fortunately, studies have shown that some D‐amino acids have antibiofilm properties. The mechanism of the antibiofilm activities of D‐amino acids is shown in Figure 4. Although the mechanism of their antibiofilm activity is not well known, it may be related to the ability of D‐amino acids to prevent initial adhesion by changing the extracellular matrix. For example, it has been reported that *Bacillus subtilis* and *Staphylococcus aureus* bind to other cells through a series of surface proteins, and several D‐amino acids prevent the colonization of these proteins on the cell surface (Hochbaum et al., 2011; Li et al., 2015). Moreover, biofilm formation increases bacterial antibiotic resistance, while the use of D‐amino acids increases the effectiveness of antibiotics due to the destruction of biofilms (Elgamoudi et al., 2020; Zilm et al., 2017).

Studies also indicate that D‐tryptophan is one of the D‐amino acids and can prevent the initial formation and further expansion of biofilms (Elgamoudi et al., 2020; Li et al., 2015) (Figure 4). In a study conducted by Elgamoudi et al. (2020), it was observed that D‐tryptophan reduces the expression of the two genes; alanine racemase (alr) and D‐Ala‐D‐Ala ligase (ddlA) in *C. Jejuni*. Both of these genes encode enzymes that participate in the metabolism of D‐alanine, which is necessary for peptidoglycan synthesis in the bacterial cell wall. Therefore, D‐tryptophan prevents biofilm formation by *C. jejuni* (Elgamoudi et al., 2020).

Several studies have proven that the use of D‐tryptophan together with other D‐amino acids has better antibiofilm activity than its application alone (Elgamoudi et al., 2020; Jia, Yang, et al., 2017; Kolodkin‐Gal et al., 2010; Leiman et al., 2013; Li et al., 2015). Interestingly, L‐amino acids, unlike D‐amino acids, increased biofilm formation because pathogens can catabolize L‐amino acids, which increases their growth and biofilm formation (Elgamoudi et al., 2020; Leiman et al., 2013). The results of the articles that investigated the antibiofilm activity of D‐tryptophan against food‐borne pathogens are summarized in Table 5. The results showed that D‐tryptophan had the highest antimicrobial activity in the first 24 h, and its effect gradually decreased and reached its lowest level after 72 h. The reason for this is that D‐tryptophan prevents initial adhesion and biofilm formation in the first 24 h. In addition. Studies have also indicated that the antibiofilm effect of D‐tryptophan increases in nutritious environments because bacterial cells have more activity and movement in these environments, which leads to a decrease in cell adhesion and biofilm formation (Elgamoudi et al., 2020; Li et al., 2015).

**TABLE 5:** Articles that have investigated the antibiofilm effect of D‐tryptophan against food‐borne pathogens.

Author	Year	Concentration of D‐Trp	Pathogen	Inhibition assay	Dispersion assay	Time	Culture medium	Ref
Elgamoudi	2020	10 mM	*C. jejuni*	48%	–	–	–	Elgamoudi et al. (2020)
Elgamoudi	2020	25 mM	*C. jejuni*	52%	–	–	–	Elgamoudi et al. (2020)
Elgamoudi	2020	10 mM	*C. jejuni*	–	42%	–	–	Elgamoudi et al. (2020)
Elgamoudi	2020	≥5 mM	*C. jejuni*	–	–	–	–	Elgamoudi et al. (2020)
Li	2015	10 mM	*C. sakazakii*	87%	–	24 h	–	Li et al. (2015)
Li	2015	10 mM	*C. sakazakii*	84%	–	48 h	–	Li et al. (2015)
Li	2015	10 mM	*C. sakazakii*	76%	–	72 h	–	Li et al. (2015)
Li	2015	–	*C. sakazakii*	–	34%	72 h + 24 h	Nutrient‐rich TBS	Li et al. (2015)
Li	2015	–	*C. sakazakii*	–	16%	72 h + 24 h	Nutrient‐poor sterile water	Li et al. (2015)

## 4 CONCLUSION

In most studies, the use of 40 Mm D‐tryptophan, along with other stress conditions, had excellent antimicrobial effects on Gram‐negative and Gram‐positive food‐borne pathogens. Among the stresses, higher temperatures (43°C, 45°C) had bactericidal effects. Therefore, it can be concluded that since D‐tryptophan is a safe natural antimicrobial compound, its use reduces the risks of chemical preservatives and, at the same time, it reduces the negative effect of thermal treatments on the nutritional value of food. D‐tryptophan also does not change the organoleptic characteristics of food, and it can be used as a bio‐preservative in the food industry.

Another antimicrobial characteristic of D‐tryptophan is its antibiofilm activity. A review of studies showed that D‐tryptophan alone and specially combined with other D‐amino acids have antibiofilm activity even in amounts of 10 Mm.

Finally, few available articles have investigated the antimicrobial activity of D‐tryptophan in food models, and since there is a wide diversity of food models, further research in this area is considered necessary.

## AUTHOR CONTRIBUTIONS

**Minoo Moghimani:** Data curation (equal); resources (equal); writing – original draft (equal). **Seyyed Mohammad Ali Noori:** Investigation (equal); methodology (equal); resources (equal); visualization (equal); writing – review and editing (equal). **Asma Afshari:** Investigation (equal); resources (equal); writing – review and editing (equal). **Mohammad Hashemi:** Conceptualization (equal); investigation (equal); project administration (equal); resources (equal); supervision (equal); writing – review and editing (equal).

## FUNDING INFORMATION

There was no funding data for this review article.

## CONFLICT OF INTEREST STATEMENT

The authors displayed no conflicts of interest.

## ETHICS STATEMENT

This study does not involve any human or animal testing.

## Data Availability

The data that support the findings of this study are available from the corresponding author upon reasonable request.
